# Responses of soil micro-eukaryotic communities to decadal drainage in a Siberian wet tussock tundra

**DOI:** 10.3389/fmicb.2023.1227909

**Published:** 2024-01-05

**Authors:** Nu Ri Myeong, Min Jung Kwon, Mathias Göckede, Binu M. Tripathi, Mincheol Kim

**Affiliations:** ^1^Korea Polar Research Institute (KOPRI), Incheon, Republic of Korea; ^2^Department of Systems Biotechnology, Chung-Ang University, Anseong, Republic of Korea; ^3^Institute of Soil Science, University of Hamburg, Hamburg, Germany; ^4^Max Planck Institute for Biogeochemistry, Jena, Germany; ^5^Division of Plant and Soil Sciences, West Virginia University, Morgantown, WV, United States

**Keywords:** soil micro-eukaryotic community, drainage treatment, permafrost thaw, climate warming, Arctic tundra

## Abstract

Climate warming holds the potential to cause extensive drying of wetlands in the Arctic, but the warming-drying effects on belowground ecosystems, particularly micro-eukaryotes, remain poorly understood. We investigated the responses of soil micro-eukaryotic communities, including fungi, protists, and microbial metazoa, to decadal drainage manipulation in a Siberian wet tundra using both amplicon and shotgun metagenomic sequencing. Our results indicate that drainage treatment increased the abundance of both fungal and non-fungal micro-eukaryotic communities, with key groups such as Ascomycota (mostly order Helotiales), Nematoda, and Tardigrada being notably abundant in drained sites. Functional traits analysis showed an increase in litter saprotrophic fungi and protistan consumers, indicating their increased activities in drained sites. The effects of drainage were more pronounced in the surface soil layer than the deeper layer, as soils dry and warm from the surface. Marked compositional shifts were observed for both communities, with fungal communities being more strongly influenced by drainage-induced vegetation change than the lowered water table itself, while the vegetation effect on non-fungal micro-eukaryotes was moderate. These findings provide insights into how belowground micro-eukaryotic communities respond to the widespread drying of wetlands in the Arctic and improve our predictive understanding of future ecosystem changes.

## 1 Introduction

Over the past few decades, the Arctic has experienced warming at a rate nearly four times faster than the global average, with an increase of 0.73°C per decade between 1979 and 2021 (Rantanen et al., 2022). This warming has significantly altered the Arctic’s terrestrial landscapes, and permafrost-based ecosystems, in particular, are undergoing substantial transformations due to the accelerated thawing of permafrost under a warmer climate (Liljedahl et al., 2016). Permafrost thaw has been demonstrated to accelerate thermo-erosion processes, acting as a trigger for the formation and expansion of thermokarst lakes. These thermokarst lakes, formed by land subsidence resulting from the extensive thawing of ice-rich permafrost soil, release large amounts of greenhouse gases such as CH_4_ and CO_2_ into the atmosphere. This emission is attributed to the rapid availability of organic matter in the freshly thawed soil, providing substance for microbial mineralization processes. As a result, it leads to a positive feedback loop amplifying climate warming (Serikova et al., 2019; In't Zandt et al., 2020). Contrary to previous understanding, recent evidence indicates that permafrost thaw can also induce extensive drying of wetlands. This drying is attributed to permafrost thaw followed by lateral drainage, where water moves along the land surface due to permafrost thawing. Consequently, this process leads to drier conditions across large permafrost areas (Liljedahl et al., 2016; Webb et al., 2022). There is a growing concern that increased drying could make peatlands more susceptible to carbon loss through enhanced decomposition of organic matter, leading to the release of more carbon into the atmosphere (Huang et al., 2021). Hence, more research is necessary to understand the drainage dynamics in permafrost regions and the effects of drying on wetland ecosystems in the Arctic.

Although warming-induced drying effects are important, microbial studies examining drainage effects under both, artificial and natural conditions, in the Arctic are limited (Kwon et al., 2016; Keuschnig et al., 2022). Bacteria and archaea have been the main focus of these studies as they are believed to have a direct relationship with methane metabolism and play significant roles in organic matter decomposition in cold regions. However, the distribution and responses of micro-eukaryotes such as fungi and protists to recent climate change in the Arctic remain poorly understood, despite their ubiquity and abundance in Arctic biomes (Malard and Pearce, 2018). Soil micro-eukaryotes are essential components of Arctic terrestrial ecosystems and play pivotal roles in biogeochemical cycles through soil food webs, interactions with plants, and nutrient uptake and fluxes. Ectomycorrhizal fungi, for example, are critical for Arctic plants to uptake nutrients and survive in nutrient-poor tundra ecosystems (Hobbie et al., 2009; Bjorbækmo et al., 2010). Furthermore, the predatory activity by micro-eukaryotes regulates carbon cycling by controlling the abundance of soil prokaryotes. Trophic shifts induced by warming were identified as one of the main controlling factors in the microbial methane cycle in a warming experiment (Tveit et al., 2015). By examining the response of the micro-eukaryotic community to drainage treatment, we can gain a more accurate and predictive understanding of how warming-induced drying affects terrestrial wetland ecosystems in the Arctic.

In this study, we investigated the impact of decadal artificial drainage on the micro-eukaryotic communities in a Siberian wet tussock tundra using both marker gene-based amplicon and shotgun metagenomic sequencing data. To better reflect the inter-sample differences in microbial abundance, community data were interpreted in a semi-quantitative manner by rescaling the read counts per sample with metagenome-derived taxa abundance information. Our previous research has shown that drainage treatment significantly altered bacterial and archaeal communities, with a more prominent effect at the surface layer (0–7.5 cm) compared to the subsurface layer (7.5–15 cm; Kwon et al., 2021). Based on this, we hypothesized that decadal drainage would also lead to significant changes in the abundance, composition, and functional traits of micro-eukaryotic communities, with a more pronounced effect on the surface layer. To explore this hypothesis, we addressed two questions: (i) What are the eukaryotic taxa and functional traits most responsive to drainage treatment? (ii) Does community-level response to drainage treatment differ between fungi and non-fungal micro-eukaryotes? Additionally, we validated our drainage manipulation results by comparing them with those of two other sites within the study area: drained but still wet (drained-wet) and naturally occurring dry (control-dry) sites.

## 2 Materials and methods

### 2.1 Site description and soil sampling

The Ambolikha research site is situated on the floodplain of the Kolyma River, adjacent to Chersky, a northeastern Siberian town in Russia. The mean annual temperature within the period 1960 to 2009 was −11°C, with mean monthly temperatures ranging from −33°C in January to +12°C in July, while the average annual precipitation was 197 mm from 1950 to 1999, with mean monthly precipitation varying between 7 mm in March and 30 mm in August (Göckede et al., 2017). The site experiences periodic inundation during the growing season due to snowmelt water and flooding of the river basin. The wet tussock tundra at the site is mainly dominated by tussock-forming sedges (*Carex appendiculata* and *C. lugens*) and a cotton grass species (*Eriophorum angustifolium*), while shrubs (*Betula exilis*, *Salix fuscescens*, and *S. pulchra*) and other *Carex* spp. are more prevalent in dry sites (Kwon et al., 2016). The soils consist of a 15–25 cm thick organic peat layer with underlying silty clay alluvial soils.

Since 2004, the ‘Drained site’ (68.6131 N, 161.3414 E) has undergone drainage through a ~ 200 m diameter circular drainage ditch (Merbold et al., 2009), resulting in a lowered water table of approximately 20 cm during the growing season. A reference study area, the ‘Control site’ (68.6167 N, 161.3497 E) is situated approximately 600 m from the drained site, and was left untreated (e.g., Göckede et al., 2019). Due to irregularities in the topography of the study area, not all plots in the drained site remained dried, and similarly, some control site plots were not fully saturated with water. Further details regarding the site description are available in Göckede et al. (2017).

In July 2014, a total of 12 soil core samples were collected: six from the drainage site and six from the control site. Samples were taken at 50-m intervals following a pre-established transect for flux chamber sites (e.g., Kwon et al., 2017). Soil cores were taken manually with a 7.6 cm diameter corer down to the permafrost table. Both the corer and the knife used to divide the soil increments were sterilized with alcohol before taking each sample. With respect to the irregularities in topography mentioned above, these 12 plots selected for soil sampling were categorized into four different groups based on the water table depth during the growing season: drained-dry, drained-wet, control-wet, and control-dry (Kwon et al., 2016). We also categorized sampling spots into two groups based on dominant vegetation type: *Eriophorum* sp. dominated and shrub-*Carex* sp. dominated (see Supplementary Table S1). It should be noted that the dominant vegetation type did not necessarily correspond with the groups based on the water table depth above. The samples had a diameter of 7.6 cm and a length of 35 cm. Each core was split into two layers, namely the organic peat layer (0–15 cm) and the alluvial layer (15–35 cm), with each layer further divided into 7.5 cm segments. Prior to homogenization in a sample bag, plant roots larger than 1 mm in diameter were removed from each sample. Subsamples of the soils were placed in Falcon tubes filled with aqueous sulfate solutions during transport, and subsequently stored at −20°C until DNA extraction. An aqueous sulfate solution (25 mM sodium citrate, ten mM EDTA, and 70 g ammonium sulfate/100 mL solution, pH 5.2; Salehi and Najafi, 2014) was used to precipitate degenerative RNases and other solubilized proteins.

### 2.2 Soil chemical properties

Regarding soil chemical properties, we provided the dataset for the upper layer (0–15 cm depth) along with the detailed analytical procedures in our previous paper (Kwon et al., 2021). In this paper, we present the results for the deeper layers below the 15 cm depth (Supplementary Table S1). The measured chemical properties include soil pH, total carbon and nitrogen content, organic carbon content, gravimetric soil water content, and sulfate concentration.

### 2.3 DNA extraction, amplicon sequencing, and community analysis

Soil DNA was extracted from 2 g of each sample using DNeasy PowerSoil Kit (QIAGEN), following the manufacturer’s instructions. Extracted DNA was purified using DNeasy PowerClean Cleanup Kit (QIAGEN). We utilized a one-step PCR approach with dual-indexed fusion primers, which include Nextera P5/P7 adapters, i5/i7 indices, and target-specific forward/reverse primers, as previously described by Comeau et al. (2017). We conducted amplicon sequencing targeting the ITS2 region for fungal communities and the V4 region of the 18S rRNA gene for non-fungal micro-eukaryotic communities. Specifically, primer pairs ITS2F-ITS4R (Op De Beeck et al., 2014) were employed for amplifying fungal ITS2 region, while primer pairs E527F-E1009R (Comeau et al., 2011) were used for targeting the eukaryotic 18S V4 region. Library preparation and amplicon sequencing were performed at the Integrated Microbiome Resource (Halifax, Canada) using the v3 chemistry of Illumina MiSeq (2 × 300 bp).

To process the ITS2 and 18S sequence data, we used the DADA2 v1.20 algorithm (Callahan et al., 2016) to infer amplicon sequence variants (ASVs). The remaining adapter and primer sequences were removed using Cutadapt v2.10 (Martin, 2011). We specifically used the DADA2 ITS pipeline workflow (v1.8) to process fungal ITS2 sequences. Trimmed reads were quality-filtered using ‘filterAndTrim’ function with the following parameters: maxN = 0, truncQ = 2, maxEE = c (Anderson, 2001; Bjorbækmo et al., 2010), and minLen = 50. Representative ASV sequences were taxonomically assigned using the IDTAXA algorithm (Murali et al., 2018), with a confidence threshold of 60%, against UNITE v9.0 (Nilsson et al., 2019) for fungal ITS2 and PR^2^ v4.14.0 (Guillou et al., 2012) for micro-eukaryotic 18S sequences. Although amplification of the ITS2 region was targeted to fungi, also some ITS2 sequences from other eukryotic groups were incidentally amplified. Their proportion was overall small and most of them were filtered out by matching them against the UNITE v9.0 (Nilsson et al., 2019). Despite these efforts, certain ITS2 sequences assigned to ‘unclassified fungi’ actually did not represent fungi. Particularly with datasets from polar regions the number of reference sequences available in public databases is rather limited (Gyeong et al., 2021). To refine the exclusion of non-fungal ITS2 sequences, we performed additional BLASTN searches against the NCBI nucleotide collection (nr/nt) (downloaded Dec 2022) for those ITS2 sequences categorized as ‘unclassified fungi’ by the UNITE database. Sequences exhibiting the closest matches to reference sequences of algae or bryophytes (≥ 97% identity and E-value ≤10^−32^) were subsequently removed from the dataset. Micro-eukaryotic 18S ASV sequences only included protistan lineages (Geisen et al., 2018), and microbial metazoa generally exhibiting a body size of <1 mm in length (Bik, 2019). The raw data from amplicon sequencing are available from the NCBI Sequence Read Archive (SRA) under the BioProject accession number PRJNA588342.

### 2.4 Cross-validation with shotgun metagenomic data and relative abundance estimation

To compare the proportion of major taxa inferred from amplicon sequencing data and shotgun metagenomic sequencing data at higher taxonomic ranks, we downloaded the shotgun metagenomic dataset from the NCBI SRA database (NCBI SRA PRJNA588342; Kwon et al., 2021). This dataset was generated using the same samples employed for amplicon sequencing in this study. Subsequently, 18S rRNA gene reads were extracted from the corresponding shotgun metagenomic data via SortMeRNA v2.1b based on SILVA the 138.1 rRNA database with default settings (Kopylova et al., 2012). The obtained 18S rRNA gene sequences were classified using the IDTAXA algorithm by matching them against the SILVA 138.1 database for fungi and the PR^2^ v4.14.0 database for micro-eukaryotes.

To more accurately reflect the relative abundance of each taxon in the samples and provide a closer representation of actual community abundances, we used a method that used weighted relative abundance values for each taxon per sample. Initially, we calculated the ratio of 18S read counts assigned to fungi and non-fungal micro-eukaryotes (protists and microbial metazoa) relative to the total number of metagenomic reads. This served as a proxy for estimating the relative abundance of fungal and non-fungal micro-eukaryotic communities, respectively. Metagenome-extracted 16S and 18S reads have previously been employed as semi-quantitative measures to represent prokaryotic and fungal lineages (Guo et al., 2016). Subsequently, we estimated the coverage of each shotgun metagenomic dataset using Nonpareil v3.4.1 with default settings (Rodriguez-R et al., 2018). The values obtained by multiplying the aforementioned ratio by 1/(sample coverage) effectively indicate the abundance of fungi and micro-eukaryotes within the metagenomic dataset, assuming that the sample coverage is ideally 1. To assign an ‘abundance weight’ to the relative proportions for each taxon in the amplicon sequencing data, we applied a weighting factor derived from these metagenome-based abundance values for fungi and micro-eukaryotes by multiplying them. This approach provides a better representation of taxonomic composition aligning more closely with the true community abundances, rather than relying solely on the percent-based relative abundance of each taxon in the amplicon dataset.

### 2.5 Inference of functional traits

The functional assignments of fungal taxa were conducted using the FungalTraits database by linking the taxonomic information of fungal ASVs to functional guilds (Põlme et al., 2020). The relative proportion of each trait was calculated using the ‘microeco’ R package (Liu et al., 2021). For non-fungal micro-eukaryotic communities, a broad classification of life history strategies was employed. The major protistan taxonomic groups were classified into three life history strategies: phototrophic (Archaeplastida, Ochrophyta), parasitic (Apicomplexa, Ichtyosporea, MALV, Peronosporomycetes, Syndiniales), and consumer (Ciliophora, Rhizaria, non-Fungi Opisthokonta, Amoebozoa, non-Ochrophyta, and non-Peronosporomycetes Stramenopiles) (Singer et al., 2021).

### 2.6 Statistical analyses

We used the ‘estimateD’ function in the ‘iNEXT’ R package (Hsieh et al., 2016) to calculate ASV richness at equal sample coverage. The pairwise Bray-Curtis dissimilarities between samples were calculated using the Hellinger-transformed ASV relative abundance matrix and visualized using principal coordinates analysis (PCoA). Each soil chemical variable was fitted onto the ordination space using the ‘envfit’ function in the vegan R package, and the significance of fitted vectors was tested based on 999 permutations (Oksanen et al., 2013). To assess the effects of the drainage treatment, soil depth, and vegetation type on the microbial community structure, we conducted a permutational multivariate analysis of variance (PERMANOVA) and *p*-values were obtained using 999 permutations (Anderson, 2001). We employed the Wilcoxon rank sum test to identify statistically significant differences in the relative abundance of taxa affected by either drainage treatment or vegetation type. All plots were created using standard functions in R v4.2.2 (R Core Team, 2021).

## 3 Results

### 3.1 Drainage effects on fungal communities

**Figure 1:**
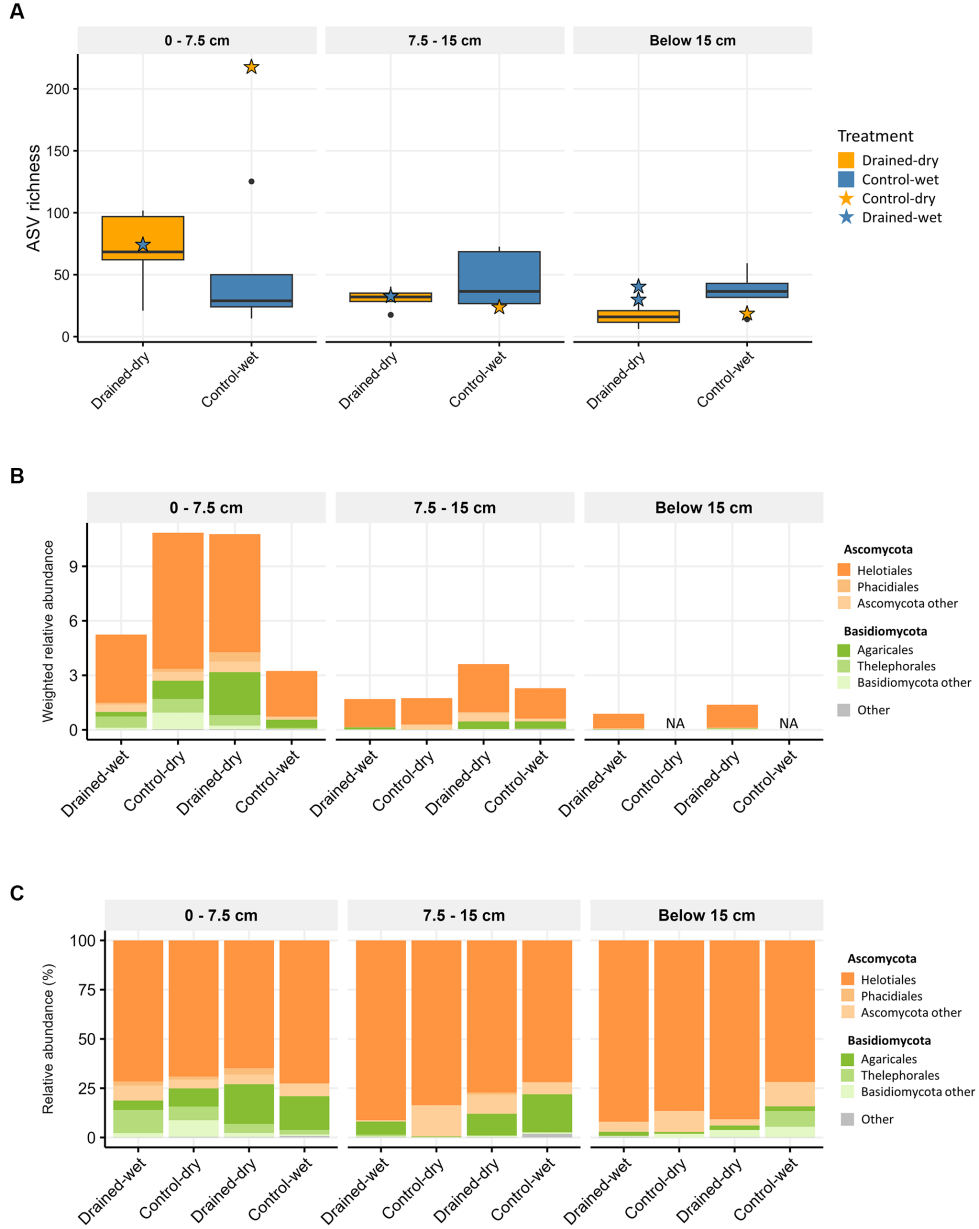
Boxplots showing fungal **(A)** ASV richness, and stacked bar plots displaying the taxonomic composition at the order level using **(B)** weighted relative abundance and **(C)** percent relative abundance. The average weighted relative abundance values for each treatment group are displayed in **(B)**. Samples with low biomass resulting in no metagenomic data available are indicated as NA in **(B)**. Only orders with relative abundance higher than 1% are presented in **(B,C)**.

Amplicon sequencing of the fungal ITS2 region generated 916,266 high-quality reads, comprising a total of 1,136 fungal ASVs. At the 0–7.5 cm depth, fungal ASV richness was on average 1.4-fold higher in drained-dry plots compared to control-wet, but the difference was not statistically significant (Wilcoxon rank sum test, *p* > 0.05; Figure 1A). No significant differences in fungal ASV richness between drained-dry and control-wet plots were observed at the lower two soil depths (all *p* > 0.05; Figure 1A). When the extent of ASV richness was compared by dominant vegetation type, a significant difference was observed at the 0–7.5 cm depth, with fungal ASV richness being on average 2.4-fold higher in shrub-*Carex* sp. dominated plots compared to *Eriophorum* sp. dominated plots (W = 32, *p* < 0.01). At both the 7.5–15 cm and below the 15 cm depths, there was no significant difference in fungal ASV richness between different vegetation types (*p* > 0.05). Additionally, no significant effects of the interaction terms (drainage x vegetation) on the ASV richness were observed at any of the soil depths (ANOVA, *p* > 0.05).

**Figure 2:**
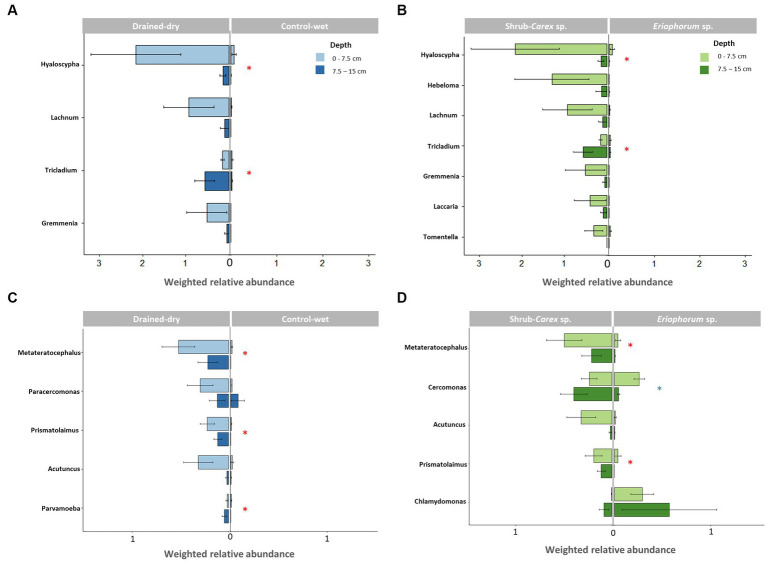
Differential abundance of dominant genera based on drainage treatment and vegetation type. **(A,B)** Represent fungal genera, while **(C,D)** depict non-fungal micro-eukaryotic genera. Weighted relative abundance values at two soil depths (0–7.5 cm and 7.5–15 cm) were used for comparison. The red asterisk indicates a significant difference in both 0–7.5 cm and 7.5–15 cm depths (*p* < 0.05), while no asterisk and blue asterisk represent a significant difference only at 0–7.5 cm and 7.5–15 cm depth, respectively.

Ascomycota and Basidiomycota were the most dominant fungal phyla, accounting for on average 84.5 and 15.1% of all fungal ITS reads, respectively. Metagenomic comparisons confirmed a strong correlation between their relative abundances across samples and those obtained from fungal 18S reads in shotgun metagenomic data (Supplementary Figure S1A). To more accurately represent the abundance of taxa in the samples, we compared the samples in a semi-quantitative manner using weighted relative abundance values. The drainage treatment led to a significant increase in the weighted relative abundance of fungi at both the 0–7.5 cm depth and 7.5–15 cm depth (all *p* < 0.05; Figure 1B). The abundance difference between treatments was more pronounced at the 0–7.5 cm depth than at the 7.5–15 cm depth. At the 0–7.5 cm depth, the weighted relative abundance of Ascomycota (mostly comprising order Helotiales) was significantly higher in drained-dry plots than in control-wet plots (*p* < 0.05; Figure 1B). The control-dry samples displayed a similar pattern to the drained-dry result, while the drained-wet samples showed a similarity to the control-wet result. On the other hand, the difference in percent relative abundance (%) at the phylum and order levels was indistinguishable between treatments at all depths (all *p* > 0.05; Figure 1C). The weighted relative abundance of Basidiomycota did not differ by drainage treatment, but significantly varied by vegetation type at the 7.5–15 cm depth, with significantly higher abundance observed in shrub-*Carex* sp. dominated plots compared to *Eriophorum* sp. dominated plots (*p* < 0.05; Supplementary Figure S2A). Among Basidiomycota orders, the relative abundance of Agaricales differed significantly depending on vegetation type at this depth (*p* < 0.05). At the genus level, genus *Hyaloscypha*, *Lachnum*, *Tricladium*, and *Gremmenia* showed a higher relative abundance in drained-dry plots than control-wet plots at the 0–7.5 cm depth (all *p* < 0.05; Figure 2A). Genus *Hebeloma*, *Laccaria*, and *Tomentella* differed in relative abundance only by vegetation type at the 0–7.5 cm depth (all *p* < 0.05; Figure 2B).

**Figure 3:**
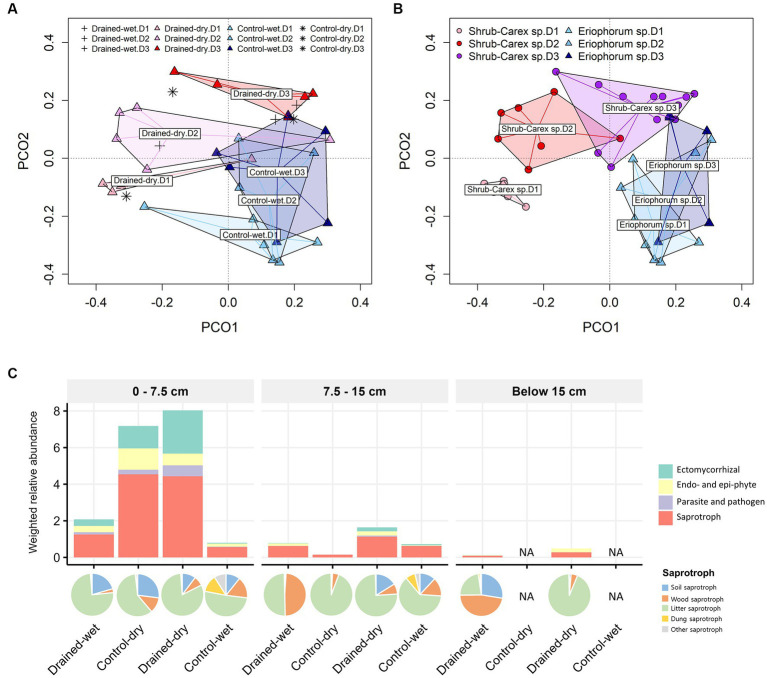
Principal coordinate analysis (PCoA) plots of fungal communities based on **(A)** drainage treatment and **(B)** vegetation type, and **(C)** functional group distribution. The primary lifestyle-based weighted relative abundance of functional groups is shown in **(C)** with the sublevel composition of saprotrophs represented by a pie chart. In **(A)**, drained-wet and control-dry samples are denoted by plus and asterisk symbols, respectively. In **(A,B)**, D1, D2, and D3 represent 0–7.5 cm, 7.5–15 cm, and below 15 cm depths, respectively.

The fungal community structure differed significantly by drainage treatment, vegetation type, and soil depth, with the vegetation effect being the strongest, followed by the treatment and soil depth effects (Table 1 and Figures 3A,B). A drainage-induced compositional shift was evident at the 0–15 cm depth (*p* < 0.01), but the drainage effect was not significant below the 15 cm depth (*p* > 0.05; Supplementary Table S2). When the vectors of soil physicochemical variables were fitted onto the ordination space, fungal community variation was associated primarily with sulfate concentration (goodness of fit, *R*^2^ = 0.53), followed by organic carbon content (*R*^2^ = 0.48) and pH (R^2^ = 0.44; Supplementary Figure S3A). The comparison sites, both control-dry and drained-wet samples, were placed within the cluster of drained-dry samples (Figure 3A).

**Table 1:** Summary of PERMANOVA partitioning results comparing effects of drainage treatment, soil depth, and vegetation type on fungal and non-fungal micro-eukaryotic community structure.

Source of variation	*df*	Fungi				Non-fungal micro-eukaryotes			
		SS	MS	Pseudo-F	P (perm)	SS	MS	Pseudo-F	P (perm)
Drainage	1	6,277	6,277	2.200	**0.001**	5,605	5,605	1.654	**0.004**
Soil depth	2	7,889	3,944	1.383	0.06	13,298	6,649	1.962	**0.001**
Vegetation	1	8,973	8,973	3.145	**0.001**	5,886	5,886	1.736	**0.003**
Drainage x Soil depth	2	4,133	2,067	0.724	0.929	6,857	3,429	1.012	0.459
Drainage x Vegetation	1	5,177	5,177	1.815	0.011	4,075	4,075	1.202	0.192
Soil depth x Vegetation	2	5,855	2,928	1.026	0.425	7,584	3,792	1.119	0.258
Drainage x Soil depth x Vegetation	2	5,580	2,790	0.978	0.5	7,361	3,681	1.086	0.312
Residual	22	62,766	2,853	–	–	74,573	3,390		
Total	33	116,990	–	–	–	133,570			

*p*-values were obtained using 999 permutations under a reduced model. SS, sum of squares; MS, mean sum of squares. The bold values indicate a statistically significant difference (*p* < 0.01) between the compared groups.

To investigate whether the drainage treatment and vegetation type led to any functional shifts in fungal communities, we assigned functional traits to each ASV sequence using the FungalTraits database. When functional traits were categorized by primary lifestyle, the drainage treatment led to a significant increase in the weighted relative abundance of litter saprotrophs (0.3 ± 0.2 in the control-wet vs. 3.6 ± 2.5 in the drained-dry) at the 0–7.5 cm depth (W = 24, *p* < 0.05; Figure 3C). An increase in the weighted relative abundance of the litter saprotrophs was also observed at the 7.5–15 cm depth, but the difference was not statistically significant (*p* > 0.05). The weighted relative abundance of ectomycorrhizal fungi did not vary by drainage treatment, but differed by vegetation type at the 0–7.5 cm depth (W = 25, *p* < 0.05).

### 3.2 Drainage effects on non-fungal micro-eukaryotic communities

**Figure 4:**
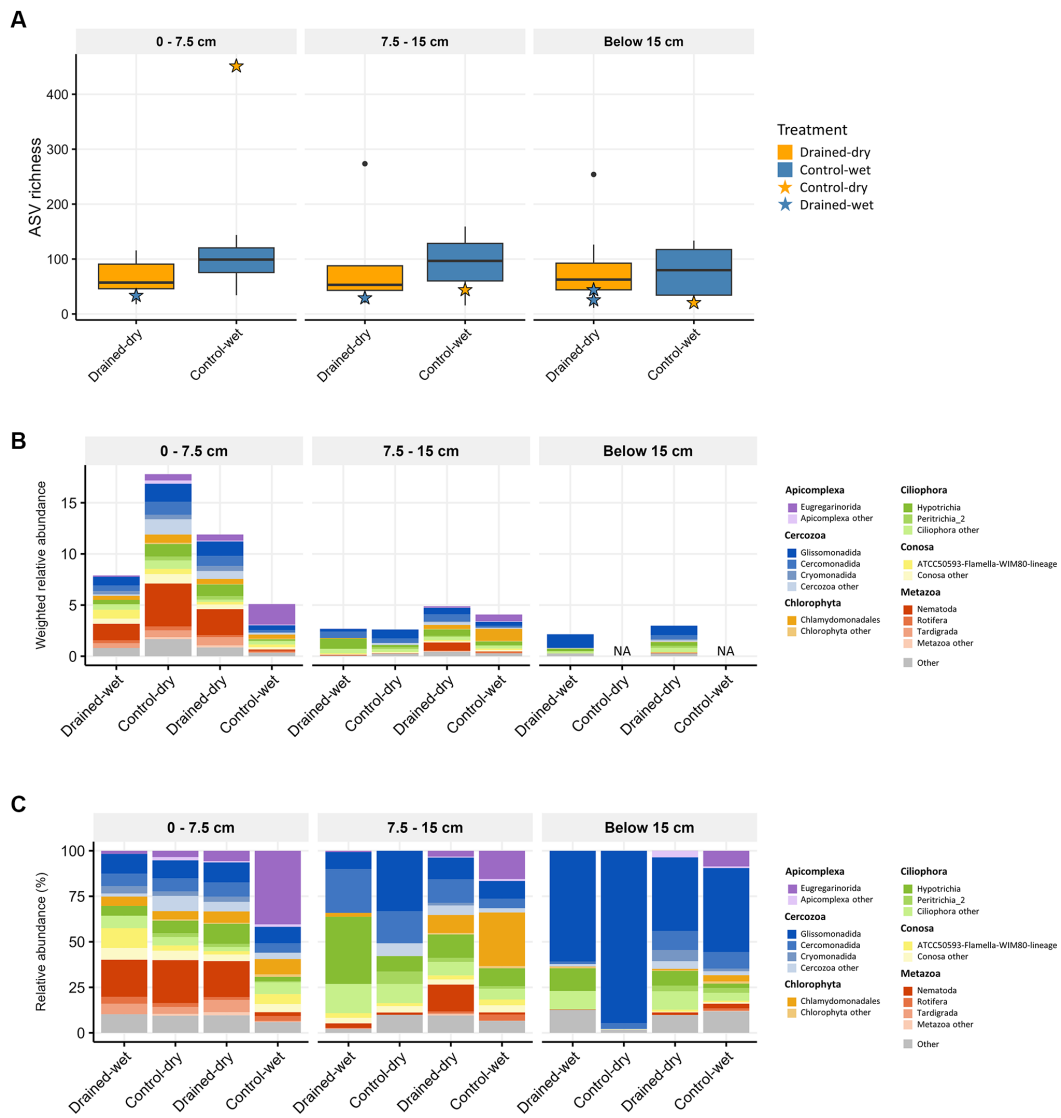
Boxplots showing non-fungal micro-eukaryotic **(A)** ASV richness, and stacked bar plots displaying the taxonomic composition at the phylum or class levels using **(B)** weighted relative abundance and **(C)** percent relative abundance. The average weighted relative abundance values for each treatment group are displayed in **(B)**. Samples with low biomass resulting in no metagenomic data available are indicated as NA in **(B)**. Only phyla or classes with relative abundance higher than 1% are presented in both **(B,C)**.

Non-fungal micro-eukaryotic communities (protists and microbial metazoa) accounted for 20.2% of the total 18S amplicon reads, and were clustered into 1,999 ASVs. Micro-eukaryotic ASV richness did not differ significantly by neither drainage treatment nor vegetation type at all soil depths (all *p* > 0.05; Figure 4A). The most abundant lineages were Cercozoa (on average 40.9%), Ciliophora (16.9%), and microbial metazoa (11.1%), which collectively accounted for 68.9% of the total 18S reads. We observed a general agreement between amplicon- and metagenome-based 18S reads in terms of the proportions of these three groups (Supplementary Figure S1B). The weighted relative abundance of non-fungal micro-eukaryotic communities significantly increased in drained-dry plots at both the 0–7.5 and 7.5–15 cm soil depths (Figure 4B). Similar to the fungal abundance, the increase was more pronounced at the 0–7.5 cm depth compared to the 7.5–15 cm depth. The weighted relative abundance of major micro-eukaryotic phyla varied between treatments, with drained-dry plots showing a significantly higher relative abundance of microbial metazoa (mostly Nematoda and Tardigrada) compared to control-wet plots at the 0–7.5 cm depth (all *p <* 0.05; Figure 4B). The similar trend was also observed in drained-wet and control-dry plots. However, the difference in percent relative abundance (%) at the phylum and class levels was indistinguishable between treatments, except for microbial metazoa (Figure 4C). At the genus level, the relative abundance of the genera *Metateratocephalus* (Nematoda), *Paracercomonas* (Cercozoa), *Prismatolaimus* (Nematoda), and *Acutuncus* (Tardigrada) was significantly higher in drained-dry plots than in control-wet plots at the 0–7.5 cm depth (all *p* < 0.05; Figure 2C). The genus *Cercomonas* (Cercozoa) showed a higher relative abundance in shrub-*Carex* sp. dominated plots only at the 7.5–15 cm depth, whereas a substantial reduction in the relative abundance of the genus *Chlamydomonas* (Chlorophyta) in shrub-*Carex* sp. dominated plots at both the 0–7.5 and 7.5–15 cm soil depths, with statistically significance observed only at the 0–7.5 depth (*p* < 0.05) (Figure 2D). The vast majority of Chlorophyta ASVs were assigned to the order Chlamydomonadales, primarily attributed to the genus *Chlamydomonas*. A smaller portion, labeled as ‘Chlorophyta other,’ consisted of unclassified Chlorophyta, orders Chaetophorales, Chlorellales, and Sphaeropleales. The relative abundance of ‘Chlorophyta other’ was less than 1% on average, and they are sporadically distributed across the samples (see Figure 4C). Due to their limited representation and a lack of a discernible response to the drainage treatment, they will not be discussed in more detail here.

**Figure 5:**
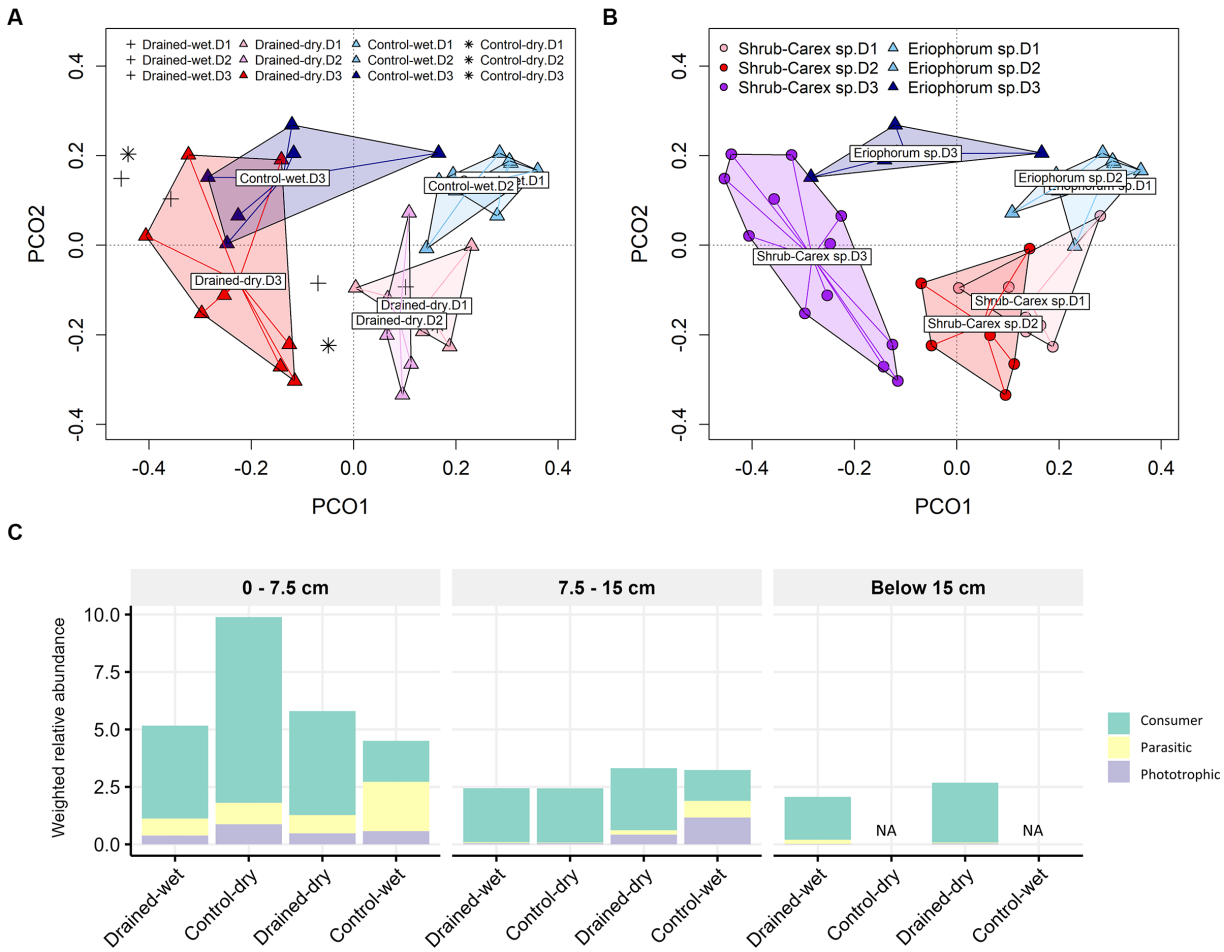
Principal coordinate analysis (PCoA) plots of non-fungal micro-eukaryotic communities based on **(A)** drainage treatment and **(B)** vegetation type, and **(C)** functional group distribution. Panel **(C)** displays the weighted relative abundance of each protistan life history strategy, namely consumer, parasite, and phototroph, averaged within each group category. In **(A)**, plus and asterisk symbols represent samples from drained-wet and control-dry plots, respectively. In **(A,B)**, D1, D2, and D3 represent 0–7.5 cm, 7.5–15 cm, and below 15 cm depths, respectively.

The non-fungal micro-eukaryotic communities were primarily structured by soil depth, followed by vegetation type and drainage treatment, with similar effect sizes (Table 1 and Figures 5A,B). The effect of drainage was statistically significant at the 0–15 cm depth (*p* < 0.01), but not below the 15 cm depth (*p* > 0.05; Supplementary Table S2). The non-fungal micro-eukaryotic community variation was associated primarily with total nitrogen content (*R*^2^ = 0.71) and organic carbon content (*R*^2^ = 0.70), followed by sulfate concentration (*R*^2^ = 0.46; Supplementary Figure S3B). Similar to fungal communities, drained-wet and control-dry samples were placed within the cluster of drained-dry samples (Figure 5A). When micro-eukaryotic communities were categorized by life history strategies (e.g., consumer, phototroph, and parasite), the weighted relative abundance of consumers significantly increased in drained-dry plots compared to control-wet plots at the 0–7.5 cm depth (Figure 5C). There was no significant increase in the weighted relative abundance of consumers at the 7.5–15 cm depth (*p* > 0.05).

## 4 Discussion

### 4.1 Effects of decadal drainage on the abundance and taxonomy of micro-eukaryotic communities

The abundance of both fungal and non-fungal micro-eukaryotic communities was significantly increased by decadal drainage. As a result of the prolonged drainage, the water table in drained-dry plots has lowered by approximately 20 cm for a decade relative to control-wet plots, which has resulted in surface soils becoming more aerobic (Kwon et al., 2016). Since eukaryotic microorganisms generally prefer aerobic conditions for growth and survival (Jaatinen et al., 2007), the higher abundance of micro-eukaryotes in drained-dry plots is not surprising. However, in contrast to eukaryotes, previous research conducted at the same site revealed lower relative abundances of bacterial and archaeal communities in drained-dry plots compared to control-wet plots (Kwon et al., 2021). Furthermore, during the growing season, heterotrophic respiration (R_h_) from soil organisms at the 0–15 cm depth was observed to be higher in drained-dry plots as compared to control-wet plots (Kwon et al., 2021). Although this study did not quantify the relative contributions of prokaryotes and micro-eukaryotes to the total R_h_, it is plausible to assume that the increased abundance of micro-eukaryotic communities has some correlation with the elevated R_h_ in drained-dry plots. It suggests that the increased activity of micro-eukaryotes in response to drying may play a significant role in the overall carbon loss from the ecosystem in dried peatlands. These findings are consistent with a study on a Polish peatland, where ecosystem respiration increased in dried sites where saprotrophic fungi became more prevalent in response to extreme drought (Jassey et al., 2018). This highlights the usefulness of abundance data for both soil eukaryotes and prokaryotes in linking microbial dynamics with heterotrophic respiration belowground.

Following a decade of drainage treatment, there was a notable increase in the abundance of order Helotiales, which corresponded to a functional transition marked by an increase in litter saprotrophic fungi. The order Helotiales comprises the most extensive yet undescribed root-associated fungal species with versatile lifestyles (Wang et al., 2006; Tedersoo et al., 2009). This increase in Helotiales aligns with previous findings that reported their prevalence in dry tundra soils of the Arctic (Dahl et al., 2017; Voříšková et al., 2019). Similarly, other peatland ecosystems have also reported an increase in saprotrophic fungi as a result of reduced water levels (Asemaninejad et al., 2017; Jassey et al., 2018). This increase in abundance may be attributed to their greater organic matter degradability and heterotrophic respiration under aerobic conditions following persistent water table drawdown. Although the relative abundance of ectomycorrhizal fungi did not vary significantly due to drainage treatment, it was found to differ according to vegetation type. The ectomycorrhizal genera *Hebeloma* and *Tomentella*, both of which were found in greater abundance in shrub-*Carex* sp. dominated plots, have been reported to form ectomycorrhizal symbiotic relationships with shrubs in the Arctic (Cripps and Eddington, 2005; Bjorbækmo et al., 2010). Therefore, the increase in shrub cover induced by lowered water levels may have facilitated root mycorrhizal colonization.

Similar to the fungal abundance, the abundance of microbial metazoa, in particular nematoda and tardigrada, also increased in drained-dry plots. It is expected that the abundance of nematodes at the surface layer in drained-dry plots would increase, as they are negatively affected by high moisture content (Kuzmin, 1976; Afzal et al., 2021). A similar finding was also reported in a drained temperate peatland (Weil et al., 2020). At the genus level, two bacterivorous nematode genera, *Metateratocephalus* and *Prismatolaimus*, were found to be more abundant in drained-dry plots. They have been reported to occur in various terrestrial and freshwater environments, including Svalbard, Taymyr, and Novaya Zemlya in Russia, in the Arctic (Holovachov, 2014). Furthermore, we observed an increase in protistan consumers at the surface layer in drained-dry plots, suggesting increased trophic interactions between protists and their prey populations. Protistan grazing has been identified as a key factor in controlling bacterial populations in peatlands (Mieczan and Tarkowska-Kukuryk, 2017). However, we could not find any significant drainage effect on the abundance of specific protistan lineages at various taxonomic ranks, except for a cercozoa genus *Paracercomonas*, which increased in drained-dry plots. Since the protistan consumer group is composed of various taxonomic lineages, the increased trophic interactions by drainage treatment could be the combined effect of multiple protistan groups. It is important to note that protists and microbial metazoa are the least known taxonomic groups in soil microbial ecology (Geisen et al., 2018; Bik, 2019), and their diversity in Arctic terrestrial ecosystems is relatively unknown compared to that in other biomes (Geisen et al., 2015). Therefore, the functional roles of these groups in dried conditions are difficult to infer due to the lack of information about their physiology and diversity. Although we did not observe a significant effect of the lowered water table, there was a notable reduction in the abundance of the green algae genus *Chlamydomonas* in response to a shift in dominant plant types. In plots dominated by shrub-*Carex* sp., this genus exhibited an average 74-fold decrease in abundance compared to those dominated by *Eriophorum* sp. *Chlamydomonas* is commonly observed in various environments, including soil (Darienko and Friedl, 2021), Arctic wetlands, and freshwater settings (Schartau et al., 2022). The reason why *Chlamydomonas* abundance was influenced more by dominant vegetation types rather than the lowered water table remains unclear. The strong correlation with plant types suggests a parallel response to the drainage treatment, akin to the delayed response often seen in the plant community transitions when faced with changes in the water table (Jukaine et al., 1995).

### 4.2 Effects of drainage treatment on alpha- and beta-diversity of micro-eukaryotic communities

In contrast to abundance responses, the drainage treatment had no significant effect on the ASV richness of both fungal and non-fungal micro-eukaryotic communities. This finding contradicts previous studies that reported increased fungal diversity as a result of lowered water tables in other peatland ecosystems (Peltoniemi et al., 2012; Jassey et al., 2018; Xue et al., 2021). This discrepancy could be explained by the duration of drainage treatment. A decade of drainage treatment may be insufficient to detect significant changes in species alpha-diversity, which may be the reason why no significant drainage effects were observed on bacterial and archaeal diversity at the same site (Kwon et al., 2021). Moreover, a study by Xue et al. (2021) found that long-term drainage (48 years) resulted in a decrease in fungal species diversity compared to short-term drainage (3 years) in a Tibetan Plateau peatland. Therefore, it is possible that changes in microbial diversity due to drainage treatment may be detectable in the longer term. The higher ASV richness of both communities in the control-dry plot compared to the control-wet plot observed at this site is the result of long-term ecosystem changes. This further supports the idea that changes in microbial alpha-diversity due to drainage treatment may require a longer observation period to detect.

Unlike the alpha-diversity results, decadal drainage led to significant shifts in the composition of both fungal and non-fungal micro-eukaryotic communities, with more striking differences observed at the surface layer than the deeper layer. The stronger compositional shift at the surface layer is expected since drainage causes the soil to dry from the surface, while deeper soils below the 15 cm depth still remain inundated. These results are consistent with those obtained for bacterial and archaeal communities studied at the same site (Kwon et al., 2021). Interestingly, we found that fungal communities were more strongly influenced by drainage-induced vegetation change than by lowered water tables, whereas both drainage and vegetation had comparable effects on non-fungal community structure. This indicates that fungal communities are less susceptible to the direct effect of a lowered water table compared to other soil micro-eukaryotes, which may be associated with their higher resistance to soil desiccation (Guhr et al., 2015). The stronger influence of host plant type on fungal communities may be attributed to the close relationship between Arctic plants and fungi, as tight associations between root-associated fungi and plants in tundra ecosystems have been reported (Hobbie et al., 2009; Bjorbækmo et al., 2010). Plant-mediated changes in fungal community composition were also reported in a warming experiment in the Arctic (Deslippe et al., 2012), and a stronger role of plant litter type than the water table in structuring fungal communities was found in a peatland ecosystem (Trinder et al., 2008). The lowered water table by decadal drainage treatment did not lead to the complete shift in plant composition at this site, as vegetation changes induced by lowered water tables in general take a longer time (Jukaine et al., 1995).

The community structures of both fungi and non-fungi in control-dry plots were similar to those in drained-dry plots, but the reverse was not true. The drained-wet was placed within the cluster of drained-dry plots, but closer to the direction of control-wet plots for both communities. This pattern was also observed in bacterial communities at the same site (Kwon et al., 2021). The dominant vegetation type in both drained-wet and dry plots was shrub-*Carex* sp. dominated, which could explain their compositional similarity, likely due to the stronger influence of vegetation type on micro-eukaryotic communities.

### 4.3 Cross-validation between amplicon and shotgun metagenomic data

The proportion of major taxa detected by shotgun metagenomic sequencing was generally consistent with those by amplicon sequencing at higher taxonomic ranks. This indicates that primer biases during the PCR reaction are not significant across samples (Oliverio et al., 2020). Moreover, this taxonomic concordance reinforces the usefulness of taxa relative abundance weighting by metagenome-derived 18S reads. This semi-quantitative approach was also useful in identifying differentially abundant taxa; significant differences in abundance were detected using weighted relative abundance values, whereas percent relative abundance failed to identify differences between drainage treatments for both communities. Therefore, shotgun metagenomic sequencing with greater depth would be useful in studies where the abundance and molecular diversity of soil micro-eukaryotes are important.

## 5 Conclusion

Our study highlights the significant impact of decadal drainage manipulation on both fungal and non-fungal micro-eukaryotic communities in an Arctic wetland ecosystem. We observed increases in the relative abundance of key groups, such as Helotiales (Ascomycota) in fungi and various taxa belonging to Nematoda and Tardigrada in non-fungal micro-eukaryotes, in drained sites. Furthermore, drainage induced functional shifts in both communities, with an increase in litter saprotrophic fungi and consumer protists. Unlike the abundance response, we did not find significant effects of drying on ASV richness for both communities. The drainage treatment led to significant compositional shifts for both communities, but the extent of community-level response differed between them. Fungal communities were more strongly influenced by drainage-induced vegetation changes rather than drainage treatment itself, whereas the vegetation effect on non-fungal micro-eukaryotic communities was relatively weak. These findings provide insights into how soil micro-eukaryotes respond to hydrological changes due to climate warming in Arctic wetland ecosystems. Integrating micro-eukaryotes into the belowground microbiome framework will enhance our understanding of the functioning of belowground ecosystems in the Arctic, where studies of soil micro-eukaryotes’ response to climate change are still limited.

## Data availability statement

The datasets presented in this study can be found in online repositories. The names of the repository/repositories and accession number(s) can be found at: https://www.ncbi.nlm.nih.gov/, PRJNA588342.

## Author contributions

NM and MKi conceived the study, performed data analysis, and wrote the manuscript with feedbacks from all authors. MG designed the experiment and maintained the experimental site. MKw collected soil samples, measured soil chemical properties, and prepared samples for DNA sequencing. BT performed metagenomic data analysis. All authors contributed to the article and approved the submitted version.

## References

[ref1] Afzal S. Nesar H. Imran Z. Ahmad W. (2021). Altitudinal gradient affect abundance, diversity and metabolic footprint of soil nematodes in Banihal-pass of Pir-Panjal mountain range. Sci. Rep. 11:16214. doi: 10.1038/s41598-021-95651-x, PMID: 34376745 PMC8355321

[ref2] Anderson M. J. (2001). A new method for non-parametric multivariate analysis of variance. Austral Ecol. 26, 32–46. doi: 10.1111/j.1442-9993.2001.01070.pp.x

[ref3] Asemaninejad A. Thorn R. G. Lindo Z. (2017). Experimental climate change modifies degradative succession in boreal peatland fungal communities. Microb. Ecol. 73, 521–531. doi: 10.1007/s00248-016-0875-9, PMID: 27744477

[ref4] Bik H. M. (2019). Microbial Metazoa Are Microbes Too. mSystems 4, e00109–e00119. doi: 10.1128/mSystems.00109-19, PMID: 31164404 PMC6584872

[ref5] Bjorbækmo M. F. M. Carlsen T. Brysting A. Vrålstad T. Høiland K. Ugland K. I.. (2010). High diversity of root associated fungi in both alpine and arctic *Dryas octopetala*. BMC Plant Biol. 10, 1–12. doi: 10.1186/1471-2229-10-244, PMID: 21070665 PMC3095326

[ref6] Callahan B. J. McMurdie P. J. Rosen M. J. Han A. W. Johnson A. J. A. Holmes S. P. (2016). DADA2: high-resolution sample inference from Illumina amplicon data. Nat. Methods 13, 581–583. doi: 10.1038/nmeth.3869, PMID: 27214047 PMC4927377

[ref7] Comeau A. M. Douglas G. M. Langille M. G. I. (2017). Microbiome helper: a custom and streamlined workflow for microbiome research. mSystems 2, e00127–e00116. doi: 10.1128/mSystems.00127-16 PMC5209531 28066818

[ref8] Comeau A. M. Li W. K. W. Tremblay J.-É. Carmack E. C. Lovejoy C. (2011). Arctic Ocean microbial community structure before and after the 2007 record sea ice minimum. PLoS One 6:e27492. doi: 10.1371/journal.pone.0027492, PMID: 22096583 PMC3212577

[ref9] Cripps C. L. Eddington L. H. (2005). Distribution of mycorrhizal types among alpine vascular plant families on the Beartooth plateau, Rocky Mountains, USA, in reference to large-scale patterns in arctic–alpine habitats. Arct. Antarct. Alp. Res. 37, 177–188. doi: 10.1657/1523-0430(2005)037[0177:DOMTAA]2.0.CO;2

[ref10] Dahl M. B. Priemé A. Brejnrod A. Brusvang P. Lund M. Nymand J.. (2017). Warming, shading and a moth outbreak reduce tundra carbon sink strength dramatically by changing plant cover and soil microbial activity. Sci. Rep. 7:16035. doi: 10.1038/s41598-017-16007-y, PMID: 29167456 PMC5700064

[ref11] Darienko T. Friedl T. (2021). 2.6 eukaryotic algal communities of rock surfaces. *Life at rock surfaces: challenged by extreme light*. Temperature Hydration Fluctuations 9:189. doi: 10.1515/9783110646467-008

[ref12] Deslippe J. R. Hartmann M. Simard S. W. Mohn W. W. (2012). Long-term warming alters the composition of Arctic soil microbial communities. FEMS Microbiol. Ecol. 82, 303–315. doi: 10.1111/j.1574-6941.2012.01350.x, PMID: 22404643

[ref13] Geisen S. Mitchell E. A. D. Adl S. Bonkowski M. Dunthorn M. Ekelund F.. (2018). Soil protists: a fertile frontier in soil biology research. FEMS Microbiol. Rev. 42, 293–323. doi: 10.1093/femsre/fuy006, PMID: 29447350

[ref14] Geisen S. Tveit A. T. Clark I. M. Richter A. Svenning M. M. Bonkowski M.. (2015). Metatranscriptomic census of active protists in soils. ISME J. 9, 2178–2190. doi: 10.1038/ismej.2015.30, PMID: 25822483 PMC4579471

[ref15] Göckede M. Kittler F. Kwon M. J. Burjack I. Heimann M. Kolle O.. (2017). Shifted energy fluxes, increased Bowen ratios, and reduced thaw depths linked with drainage-induced changes in permafrost ecosystem structure. Cryosphere 11, 2975–2996. doi: 10.5194/tc-11-2975-2017

[ref16] Göckede M. Min Jung K. Kittler F. Heimann M. Zimov N. Zimov S. (2019). Negative feedback processes following drainage slow down permafrost degradation. Glob. Chang. Biol. 25, 3254–3266. doi: 10.1111/gcb.14744, PMID: 31241797 PMC6851682

[ref17] Guhr A. Borken W. Spohn M. Matzner E. (2015). Redistribution of soil water by a saprotrophic fungus enhances carbon mineralization. Proc. Natl. Acad. Sci. 112, 14647–14651. doi: 10.1073/pnas.151443511, PMID: 26554004 PMC4664368

[ref18] Guillou L. Bachar D. Audic S. Bass D. Berney C. Bittner L.. (2012). The Protist ribosomal reference database (PR2): a catalog of unicellular eukaryote small sub-unit rRNA sequences with curated taxonomy. Nucleic Acids Res. 41, D597–D604. doi: 10.1093/nar/gks1160, PMID: 23193267 PMC3531120

[ref19] Guo J. Cole J. R. Zhang Q. Brown C. T. Tiedje J. M. (2016). Microbial community analysis with ribosomal gene fragments from shotgun metagenomes. Appl. Environ. Microbiol. 82, 157–166. doi: 10.1128/AEM.02772-15, PMID: 26475107 PMC4702641

[ref20] Gyeong H. Hyun C. U. Kim S. C. Tripathi B. M. Yun J. Kim J.. (2021). Contrasting early successional dynamics of bacterial and fungal communities in recently deglaciated soils of the maritime Antarctic. Mol. Ecol. 30, 4231–4244. doi: 10.1111/mec.16054, PMID: 34214230

[ref21] Hobbie J. E. Hobbie E. A. Drossman H. Conte M. Weber J. C. Shamhart J.. (2009). Mycorrhizal fungi supply nitrogen to host plants in Arctic tundra and boreal forests: 15N is the key signal. Can. J. Microbiol. 55, 84–94. doi: 10.1139/W08-127, PMID: 19190704

[ref22] Holovachov O. (2014). Nematodes from terrestrial and freshwater habitats in the Arctic. Biodivers. Data J. 2:e1165. doi: 10.3897/BDJ.2.e1165, PMID: 25197239 PMC4152839

[ref23] Hsieh T. C. Ma K. H. Chao A. (2016). iNEXT: an R package for rarefaction and extrapolation of species diversity (H ill numbers). Methods Ecol. Evol. 7, 1451–1456. doi: 10.1111/2041-210X.12613

[ref24] Huang Y. Ciais P. Luo Y. Zhu D. Wang Y. Qiu C.. (2021). Tradeoff of CO2 and CH4 emissions from global peatlands under water-table drawdown. Nat. Clim. Chang. 11, 618–622. doi: 10.1038/s41558-021-01059-w

[ref25] In't Zandt M. H. Liebner S. Welte C. U. (2020). Roles of thermokarst lakes in a warming world. Trends Microbiol. 28, 769–779. doi: 10.1016/j.tim.2020.04.002, PMID: 32362540

[ref26] Jaatinen K. Fritze H. Laine J. Laiho R. (2007). Effects of short-and long-term water-level drawdown on the populations and activity of aerobic decomposers in a boreal peatland. Glob. Chang. Biol. 13, 491–510. doi: 10.1111/j.1365-2486.2006.01312.x

[ref27] Jassey V. E. J. Reczuga M. K. Zielińska M. Słowińska S. Robroek B. J. M. Mariotte P.. (2018). Tipping point in plant–fungal interactions under severe drought causes abrupt rise in peatland ecosystem respiration. Glob. Chang. Biol. 24, 972–986. doi: 10.1111/gcb.13928, PMID: 28991408

[ref28] Jukaine Laine J. Vasander H. Laiho R. (1995). Long-term effects of water level drawdown on the vegetation of drained pine mires in southern Finland. J. Appl. Ecol. 32:785. doi: 10.2307/2404818

[ref29] Keuschnig C. Larose C. Rudner M. Pesqueda A. Doleac S. Elberling B.. (2022). Reduced methane emissions in former permafrost soils driven by vegetation and microbial changes following drainage. Glob. Chang. Biol. 28, 3411–3425. doi: 10.1111/gcb.16137, PMID: 35285570 PMC9314937

[ref30] Kopylova E. Noé L. Touzet H. (2012). SortMeRNA: fast and accurate filtering of ribosomal RNAs in metatranscriptomic data. Bioinformatics 28, 3211–3217. doi: 10.1093/bioinformatics/bts611, PMID: 23071270

[ref31] Kuzmin L. L. (1976). Free-living nematodes in the tundra of western Taimyr. Oikos 27:501. doi: 10.2307/3543469

[ref32] Kwon M. J. Beulig F. Ilie I. Wildner M. Kusel K. Merbold L.. (2017). Plants, microorganisms, and soil temperatures contribute to a decrease in methane fluxes on a drained Arctic floodplain. Glob. Chang. Biol. 23, 2396–2412. doi: 10.1111/gcb.13558, PMID: 27901306

[ref33] Kwon M. J. Heimann M. Kolle O. Luus K. A. Schuur E. A. Zimov N.. (2016). Long-term drainage reduces CO_2_ uptake and increases CO_2_ emission on a Siberian floodplain due to shifts in vegetation community and soil thermal characteristics. Biogeosciences 13, 4219–4235. doi: 10.5194/bg-13-4219-2016

[ref34] Kwon M. J. Tripathi B. M. Goeckede M. Shin S. C. Myeong N. R. Lee Y. K.. (2021). Disproportionate microbial responses to decadal drainage on a Siberian floodplain. Glob. Chang. Biol. 27, 5124–5140. doi: 10.1111/gcb.15785, PMID: 34216067

[ref35] Liljedahl A. K. Boike J. Daanen R. P. Fedorov A. N. Frost G. V. Grosse G.. (2016). Pan-Arctic ice-wedge degradation in warming permafrost and its influence on tundra hydrology. Nat. Geosci. 9, 312–318. doi: 10.1038/ngeo2674

[ref36] Liu C. Cui Y. Li X. Yao M. (2021). Microeco: an R package for data mining in microbial community ecology. FEMS Microbiol. Ecol. 97:fiaa255. doi: 10.1093/femsec/fiaa255, PMID: 33332530

[ref37] Malard L. A. Pearce D. A. (2018). Microbial diversity and biogeography in Arctic soils. Environ. Microbiol. Rep. 10, 611–625. doi: 10.1111/1758-2229.12680, PMID: 30028082

[ref38] Martin M. (2011). Cutadapt removes adapter sequences from high-throughput sequencing reads. EMBnet. J. 17, 10–12. doi: 10.14806/ej.17.1.200

[ref39] Merbold L. Kutsch W. L. Corradi C. Kolle O. Rebmann C. Stoy P. C.. (2009). Artificial drainage and associated carbon fluxes (CO2/CH4) in a tundra ecosystem. Glob. Chang. Biol. 15, 2599–2614. doi: 10.1111/j.1365-2486.2009.01962.x

[ref40] Mieczan T. Tarkowska-Kukuryk M. (2017). Microbial communities as environmental indicators of ecological disturbance in restored carbonate fen—results of 10 years of studies. Microb. Ecol. 74, 384–401. doi: 10.1007/s00248-017-0957-3, PMID: 28265694

[ref41] Murali A. Bhargava A. Wright E. S. (2018). IDTAXA: a novel approach for accurate taxonomic classification of microbiome sequences. Microbiome 6, 140–114. doi: 10.1186/s40168-018-0521-5, PMID: 30092815 PMC6085705

[ref42] Nilsson R. H. Larsson K.-H. Taylor A. F. S. Palme J. Jeppesen T. S. Schigel D.. (2019). The UNITE database for molecular identification of fungi: handling dark taxa and parallel taxonomic classifications. Nucleic Acids Res. 47, D259–D264. doi: 10.1093/nar/gky1022, PMID: 30371820 PMC6324048

[ref43] Oksanen J. Simpson G. L. Blanchet F. G. Kindt R. Legendre P. Minchin P. R.. (2013). Package ‘vegan’: Community ecology package, version 2, 1–295.

[ref44] Oliverio A. M. Geisen S. Delgado-Baquerizo M. Maestre F. T. Turner B. L. Fierer N. (2020). The global-scale distributions of soil protists and their contributions to belowground systems. Sci. Adv. 6,:eaax8787. doi: 10.1126/sciadv.aax8787, PMID: 32042898 PMC6981079

[ref45] Op De Beeck M. Lievens B. Busschaert P. Declerck S. Vangronsveld J. Colpaert J. V. (2014). Comparison and validation of some ITS primer pairs useful for fungal metabarcoding studies. PLoS One 9:e97629. doi: 10.1371/journal.pone.0097629, PMID: 24933453 PMC4059633

[ref46] Peltoniemi K. Straková P. Fritze H. Iráizoz P. A. Pennanen T. Laiho R. (2012). How water-level drawdown modifies litter-decomposing fungal and actinobacterial communities in boreal peatlands. Soil Biol. Biochem. 51, 20–34. doi: 10.1016/j.soilbio.2012.04.013

[ref47] Põlme S. Abarenkov K. Henrik Nilsson R. Lindahl B. D. Clemmensen K. E. Kauserud H.. (2020). FungalTraits: a user-friendly traits database of fungi and fungus-like stramenopiles. Fungal Divers. 105, 1–16. doi: 10.1007/s13225-020-00466-2

[ref48] R Core Team. (2021). R: a language and environment for statistical computing; R Core team: Vienna, Austria, 2022. Available at: www.r-project.org (Accessed February 17, 2022).

[ref49] Rantanen M. Karpechko A. Y. Lipponen A. Nordling K. Hyvärinen O. Ruosteenoja K.. (2022). The Arctic has warmed nearly four times faster than the globe since 1979. Commun. Earth Environ. 3:168. doi: 10.1038/s43247-022-00498-3

[ref50] Rodriguez-R L. M. Gunturu S. Tiedje J. M. Cole J. R. Konstantinidis K. T. (2018). Nonpareil 3: fast estimation of metagenomic coverage and sequence diversity. mSystems 3, e00039–e00018. doi: 10.1128/mSystems.00039-18 29657970 PMC5893860

[ref51] Salehi Z. Najafi M. (2014). RNA preservation and stabilization. Biochem. Physiol. 3:2. doi: 10.4172/2168-9652.1000126

[ref52] Schartau A. K. Mariash H. L. Christoffersen K. S. Bogan D. Dubovskaya O. P. Fefilova E. B.. (2022). First circumpolar assessment of Arctic freshwater phytoplankton and zooplankton diversity: spatial patterns and environmental factors. Freshw. Biol. 67, 141–158. doi: 10.1111/fwb.13783

[ref53] Serikova S. Pokrovsky O. S. Laudon H. Krickov I. V. Lim A. G. Manasypov R. M.. (2019). High carbon emissions from thermokarst lakes of Western Siberia. Nat. Commun. 10:1552. doi: 10.1038/s41467-019-09592-1, PMID: 30948722 PMC6449335

[ref54] Singer D. Seppey C. V. W. Lentendu G. Dunthorn M. Bass D. Belbahri L.. (2021). Protist taxonomic and functional diversity in soil, freshwater and marine ecosystems. Environ. Int. 146:106262. doi: 10.1016/j.envint.2020.106262, PMID: 33221595

[ref55] Tedersoo L. Pärtel K. Jairus T. Gates G. Põldmaa K. Tamm H. (2009). Ascomycetes associated with ectomycorrhizas: molecular diversity and ecology with particular reference to the Helotiales. Environ. Microbiol. 11, 3166–3178. doi: 10.1111/j.1462-2920.2009.02020.x, PMID: 19671076

[ref56] Trinder C. J. Johnson D. Artz R. R. E. (2008). Interactions among fungal community structure, litter decomposition and depth of water table in a cutover peatland. FEMS Microbiol. Ecol. 64, 433–448. doi: 10.1111/j.1574-6941.2008.00487.x, PMID: 18430005

[ref57] Tveit A. T. Urich T. Frenzel P. Svenning M. M. (2015). Metabolic and trophic interactions modulate methane production by Arctic peat microbiota in response to warming. Proc. Natl. Acad. Sci. 112, E2507–E2516. doi: 10.1073/pnas.14207971, PMID: 25918393 PMC4434766

[ref58] Voříšková J. Elberling B. Priemé A. (2019). Fast response of fungal and prokaryotic communities to climate change manipulation in two contrasting tundra soils. Environ. Microb. 14, 6–15. doi: 10.1186/s40793-019-0344-4, PMID: 33902718 PMC7989089

[ref59] Wang Z. Johnston P. R. Takamatsu S. Spatafora J. W. Hibbett D. S. (2006). Toward a phylogenetic classification of the Leotiomycetes based on rDNA data. Mycologia 98, 1065–1075. doi: 10.1080/15572536.2006.11832634, PMID: 17486981

[ref60] Webb E. E. Liljedahl A. K. Cordeiro J. A. Loranty M. M. Witharana C. Lichstein J. W. (2022). Permafrost thaw drives surface water decline across lake-rich regions of the Arctic. Nat. Clim. Chang. 12, 841–846. doi: 10.1038/s41558-022-01455-w

[ref61] Weil M. Wang H. Bengtsson M. Köhn D. Günther A. Jurasinski G.. (2020). Long-term rewetting of three formerly drained peatlands drives congruent compositional changes in pro-and eukaryotic soil microbiomes through environmental filtering. Microorganisms 8:550. doi: 10.3390/microorganisms8040550, PMID: 32290343 PMC7232337

[ref62] Xue D. Liu T. Chen H. Liu J. Hu J. Liu L. (2021). Fungi are more sensitive than bacteria to drainage in the peatlands of the Zoige plateau. Ecol. Indic. 124:107367. doi: 10.1016/j.ecolind.2021.107367

