# Supplementary material for: Responses of soil micro-eukaryotic communities to decadal drainage in a Siberian wet tussock tundra
Source: Front Microbiol. 2024 Jan 5;14:1227909. doi: 10.3389/fmicb.2023.1227909 (PMC10797069; doi:10.3389/fmicb.2023.1227909)
Supplement: Supplementary file 1 [file Data_Sheet_1.docx]

***Supplementary Material***

**Responses of soil micro-eukaryotic communities to decadal drainage in a Siberian wet tussock tundra**

**Nu Ri Myeong, Minjung Kwon, Mathias Göckede, Binu M. Tripathi, and Mincheol Kim^*^**

*** Correspondence:** Mincheol Kim: mincheol@kopri.re.kr

**Supplementary Table 1. Descriptions of soil samples and associated metadata.**

| Sample ID | Transect chamber | Treatment | Water table depth | Soil depth (cm) | Soil type | Dominant vegetation type | Soil pH | C:N ratio | Total C | Total N | Water content | Organic carbon | Sulfate |
| --- | --- | --- | --- | --- | --- | --- | --- | --- | --- | --- | --- | --- | --- |
|  |  |  |  |  |  |  |  |  | % | % | %, per wet g | %, per dry g | µg per dry g |
| dw.1 | T1_0 | drained-wet | wet | 0-7.5 | organic | Shrub-*Carex* sp. dominated | 5.40 | 18.920 | 35.007 | 1.850 | 56.352 | 35.211 | 103.033 |
| dw.2 | T1_0 | drained-wet | wet | 7.5-15 | organic | Shrub-*Carex* sp. dominated | 4.80 | 17.464 | 34.887 | 1.998 | 50.630 | 36.596 | 44.713 |
| dw.3 | T1_0 | drained-wet | wet | 15-22.5 | mineral | Shrub-*Carex* sp. dominated | 4.49 | 15.674 | 5.744 | 0.366 | 13.173 | 9.402 | 14.602 |
| dw.4 | T1_0 | drained-wet | wet | 22.5-28 | mineral | Shrub-*Carex* sp. dominated | 4.57 | 15.568 | 11.081 | 0.712 | 33.821 | 19.613 | 21.852 |
| dd.5 | T1_2 | drained-dry | dry | 0-7.5 | organic | Shrub-*Carex* sp. dominated | 5.09 | 18.049 | 37.283 | 2.066 | 61.905 | 35.417 | 258.383 |
| dd.6 | T1_2 | drained-dry | dry | 7.5-15 | organic | Shrub-*Carex* sp. dominated | 4.77 | 15.923 | 35.472 | 2.228 | 59.173 | 36.564 | 58.380 |
| dd.7 | T1_2 | drained-dry | dry | 15-22.5 | organic | Shrub-*Carex* sp. dominated | 4.64 | 15.154 | 29.550 | 1.950 | 49.827 | 32.414 | 49.897 |
| dd.8 | T1_4 | drained-dry | dry | 0-7.5 | organic | Shrub-*Carex* sp. dominated | 5.00 | 17.903 | 38.703 | 2.162 | 62.397 | 33.516 | 172.589 |
| dd.9 | T1_4 | drained-dry | dry | 7.5-15 | organic | Shrub-*Carex* sp. dominated | 4.75 | 18.340 | 38.760 | 2.113 | 60.321 | 33.838 | 72.109 |
| dd.10 | T1_4 | drained-dry | dry | 15-24 | organic | Shrub-*Carex* sp. dominated | 4.54 | 16.600 | 33.569 | 2.022 | 54.463 | 33.200 | 53.613 |
| dd.11 | T1_4 | drained-dry | dry | 24-33.5 | mineral | Shrub-*Carex* sp. dominated | 4.49 | 16.197 | 3.897 | 0.241 | 9.829 | 6.477 | 13.166 |
| dd.12 | T1_6 | drained-dry | dry | 0-7.5 | organic | Shrub-*Carex* sp. dominated | 5.05 | 15.565 | 32.514 | 2.089 | 59.298 | 30.964 | 161.311 |
| dd.13 | T1_6 | drained-dry | dry | 7.5-15 | organic | Shrub-*Carex* sp. dominated | 4.52 | 16.621 | 34.677 | 2.086 | 61.000 | 33.761 | 59.631 |
| dd.14 | T1_6 | drained-dry | dry | 15-22 | organic | Shrub-*Carex* sp. dominated | 4.65 | 15.014 | 23.371 | 1.557 | 47.532 | 28.571 | 31.084 |
| dd.15 | T1_6 | drained-dry | dry | 22-34 | mineral | Shrub-*Carex* sp. dominated | 4.62 | 14.650 | 3.001 | 0.205 | 6.471 | 4.778 | 8.062 |
| dd.16 | T1_8 | drained-dry | dry | 0-7.5 | organic | Shrub-*Carex* sp. dominated | 5.05 | 16.074 | 33.404 | 2.078 | 55.378 | 36.161 | 127.079 |
| dd.17 | T1_8 | drained-dry | dry | 7.5-15 | organic | Shrub-*Carex* sp. dominated | 4.65 | 16.011 | 33.101 | 2.067 | 52.838 | 36.929 | 67.291 |
| dd.18 | T1_8 | drained-dry | dry | 15-21 | organic | Shrub-*Carex* sp. dominated | 4.58 | 17.162 | 25.640 | 1.494 | 45.122 | 32.222 | 37.405 |
| dd.19 | T1_8 | drained-dry | dry | 21-28 | mineral | Shrub-*Carex* sp. dominated | 4.60 | 15.990 | 2.790 | 0.175 | 7.447 | 6.240 | 9.042 |
| dd.20 | T1_10 | drained-dry | dry | 0-7.5 | organic | *Eriophorum* sp. dominated | 4.49 | 16.785 | 32.115 | 1.913 | 53.460 | 33.457 | 62.964 |
| dd.21 | T1_10 | drained-dry | dry | 7.5-15 | organic | *Eriophorum* sp. dominated | 4.71 | 14.647 | 17.870 | 1.220 | 17.121 | 10.420 | 38.026 |
| dd.22 | T1_10 | drained-dry | dry | 15-24 | mineral | *Eriophorum* sp. dominated | 4.50 | 16.187 | 2.973 | 0.184 | 12.433 | 6.565 | 11.389 |
| cw.23 | T2_0 | control-wet | wet | 0-7.5 | organic | Shrub-*Carex* sp. dominated | 4.79 | 17.347 | 15.963 | 0.920 | 45.708 | 29.487 | 135.722 |
| cw.24 | T2_0 | control-wet | wet | 7.5-18 | organic | Shrub-*Carex* sp. dominated | 4.75 | 15.462 | 28.733 | 1.858 | 47.007 | 31.561 | 82.464 |
| cw.25 | T2_0 | control-wet | wet | 18-25.5 | mineral | Shrub-*Carex* sp. dominated | 4.54 | 18.531 | 3.563 | 0.192 | 11.311 | 6.285 | 8.143 |
| cw.26 | T2_0 | control-wet | wet | 25.5-32 | mineral | Shrub-*Carex* sp. dominated | 4.79 | 18.431 | 4.594 | 0.249 | 13.810 | 7.336 | 9.664 |
| cw.27 | T2_2 | control-wet | wet | 0-7.5 | organic | *Eriophorum* sp. dominated | 4.62 | 16.264 | 25.444 | 1.564 | 62.821 | 26.108 | 80.132 |
| cw.28 | T2_2 | control-wet | wet | 7.5-12 | organic | *Eriophorum* sp. dominated | 4.58 | 16.507 | 26.290 | 1.593 | 62.525 | 23.370 | NA |
| cw.29 | T2_2 | control-wet | wet | 12-15 | mineral | *Eriophorum* sp. dominated | NA | 14.938 | 2.024 | 0.135 | 29.813 | 8.252 | NA |
| cd.30 | T2_4 | control-dry | dry | 0-7.5 | organic | Shrub-*Carex* sp. dominated | 4.57 | 16.479 | 27.395 | 1.662 | 55.596 | 32.114 | 95.892 |
| cd.31 | T2_4 | control-dry | dry | 7.5-17 | organic | Shrub-*Carex* sp. dominated | 4.58 | 17.223 | 25.650 | 1.489 | 48.824 | 28.352 | 46.602 |
| cd.32 | T2_4 | control-dry | dry | 17-23.5 | mineral | Shrub-*Carex* sp. dominated | 4.63 | 16.208 | 3.446 | 0.213 | 7.042 | 5.492 | 11.449 |
| cw.34 | T2_8 | control-wet | wet | 0-7.5 | organic | *Eriophorum* sp. dominated | 4.80 | 16.032 | 22.191 | 1.384 | 59.187 | 23.377 | 101.211 |
| cw.35 | T2_8 | control-wet | wet | 7.5-12 | organic | *Eriophorum* sp. dominated | NA | 15.722 | 20.361 | 1.295 | 37.625 | 15.282 | NA |
| cw.36 | T2_8 | control-wet | wet | 12-23 | mineral | *Eriophorum* sp. dominated | 4.53 | 16.042 | 2.817 | 0.176 | 13.333 | 6.346 | 10.450 |
| cw.37 | T2_6 | control-wet | wet | 0-7.5 | organic | *Eriophorum* sp. dominated | 4.58 | 16.204 | 30.470 | 1.880 | 62.097 | 26.596 | 78.175 |
| cw.38 | T2_6 | control-wet | wet | 7.5-10.5 | organic | *Eriophorum* sp. dominated | NA | 14.757 | 26.547 | 1.799 | 55.852 | 28.372 | NA |
| cw.39 | T2_6 | control-wet | wet | 10.5-18 | mineral | *Eriophorum* sp. dominated | 4.64 | 16.017 | 3.449 | 0.215 | 9.655 | 5.344 | 11.149 |
| cw.40 | T2_10 | control-wet | wet | peat | organic | *Eriophorum* sp. dominated | 4.77 | 14.705 | 20.016 | 1.361 | 62.114 | 24.034 | 58.755 |
| cw.41 | T2_10 | control-wet | wet | mineral | mineral | *Eriophorum* sp. dominated | 4.77 | 14.772 | 2.951 | 0.200 | 11.876 | 6.055 | 13.027 |

**Supplementary Table 2. Summary of PERMANOVA partitioning results comparing effects of drainage treatment, and vegetation type on fungal and non-fungal micro-eukaryotic community structure at different soil depths.**

| Soil depth | Source of variation | *df* | Fungi | | | |  | Non-fungal micro-eukaryotes | | | |
| --- | --- | --- | --- | --- | --- | --- | --- | --- | --- | --- | --- |
|  |  |  | SS | MS | Pseudo-F | P (perm) |  | SS | MS | Pseudo-F | P (perm) |
| 0−15cm depth | Drainage | 1 | 4,759 | 4,759 | 1.706 | **0.003** |  | 4,997 | 4,997 | 1.492 | **0.009** |
|  | Vegetation | 1 | 7,577 | 7,577 | 2.716 | **0.001** |  | 4,762 | 4,762 | 1.422 | 0.021 |
|  | Drainage x Vegetation | 1 | 4,157 | 4,157 | 1.49 | 0.039 |  | 4,230 | 4,230 | 1.263 | 0.09 |
|  | Residual | 16 | 44,635 | 2,790 |  |  |  | 53,580 | 3,349 |  |  |
|  | Total | 19 | 65,967 |  |  |  |  | 71,494 |  |  |  |
| Below 15cm depth | Drainage | 1 | 4,202 | 4,202 | 1.445 | 0.075 |  | 4,635 | 4,635 | 1.350 | 0.089 |
|  | Vegetation | 1 | 4,379 | 4,379 | 1.506 | 0.038 |  | 5,510 | 5,510 | 1.605 | 0.021 |
|  | Drainage x Vegetation | 1 | 4,181 | 4,181 | 1.438 | 0.081 |  | 3,473 | 3,473 | 1.012 | 0.491 |
|  | Residual | 10 | 29,084 | 2,908 |  |  |  | 34,332 | 3,433 |  |  |
|  | Total | 13 | 42,760 |  |  |  |  | 48,714 |  |  |  |

*p*-values were obtained using 999 permutations under a reduced model. Abbreviations: SS, sum of squares; MS, mean sum of squares.


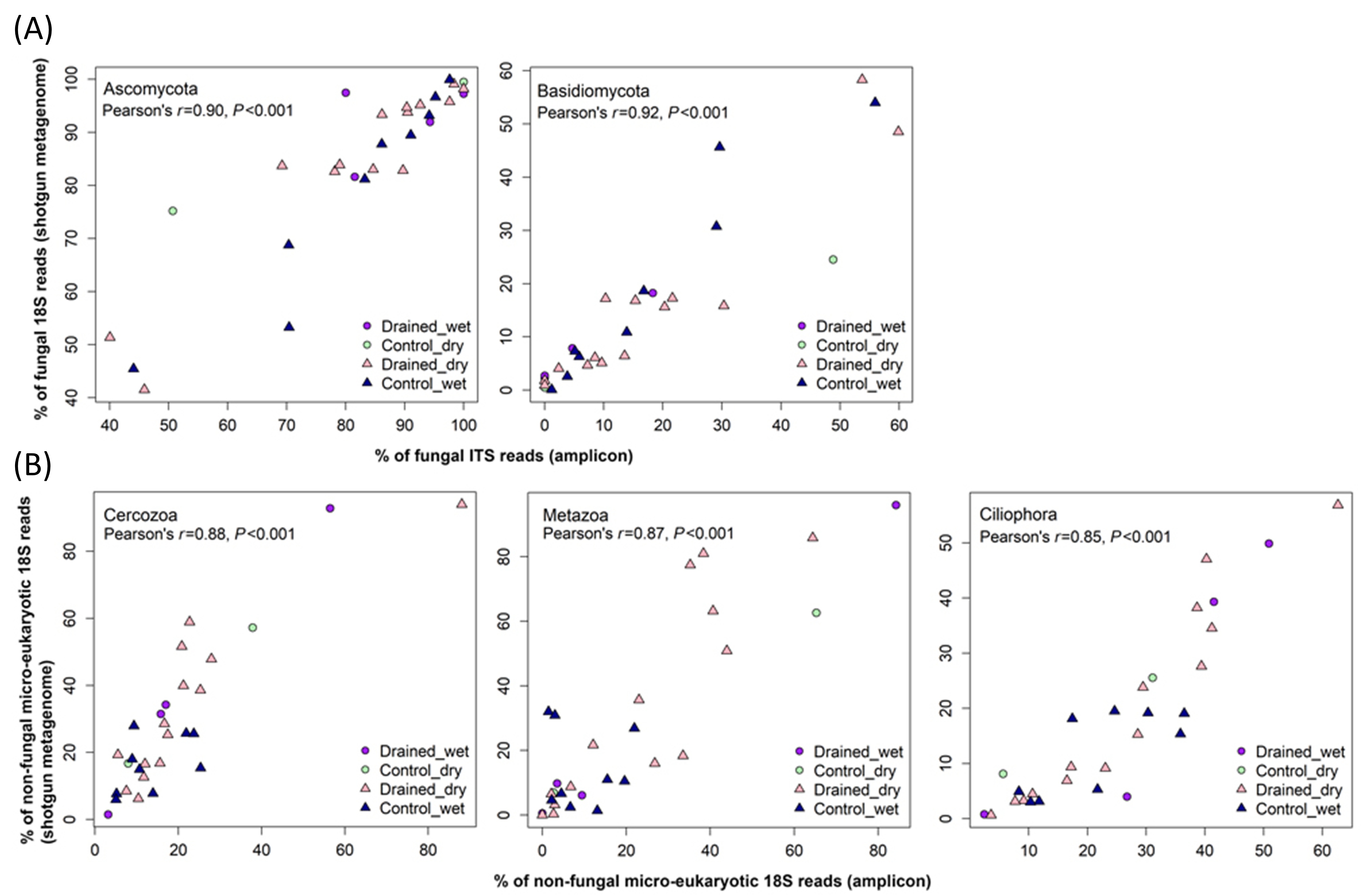


**Supplementary Figure 1. Comparisons of the percent relative abundances of dominant (a) fungal and (B) non-fungal micro-eukaryotic phyla between datasets generated by amplicon and shotgun metagenomic sequencing.** Fungi were compared using ITS reads from amplicon sequencing and 18S reads from metagenomic sequencing. Non-fungi were compared using 18S reads from both sequencing methods.


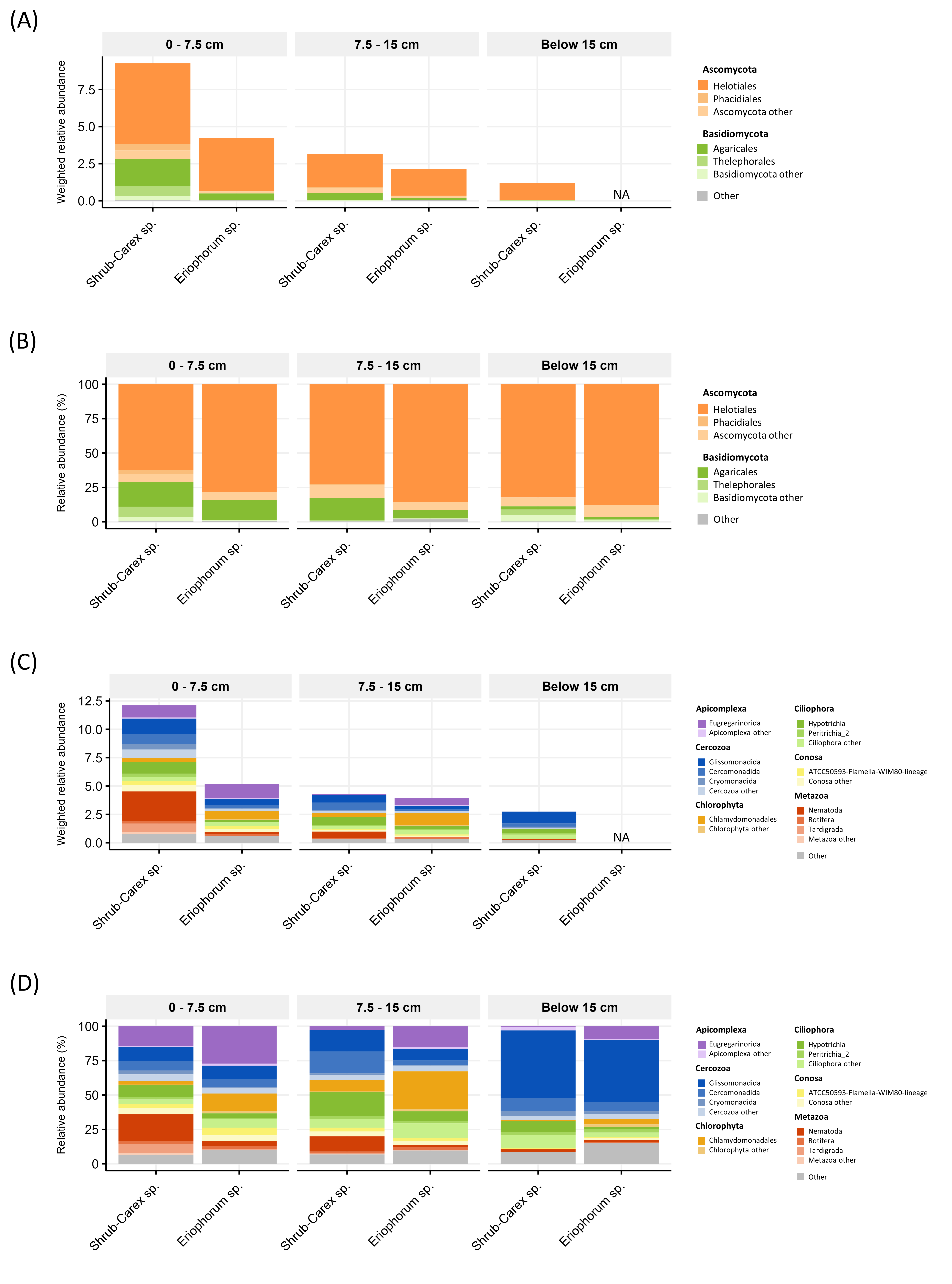


**Supplementary Figure 2. Taxonomic composition comparison of fungal and non-fungal micro-eukaryotic communities at the order level for fungi (panels A and B) and at the phylum or class level for non-fungal micro-eukaryotes (panels C and D) in two distinct vegetation types.** The relative abundance was shown as weighted relative abundance in panels (A) and (C), and as percent relative abundance (%) in panels (B) and (D). Samples with low biomass resulting in no metagenomic data available are indicated as NA in (A) and (C).


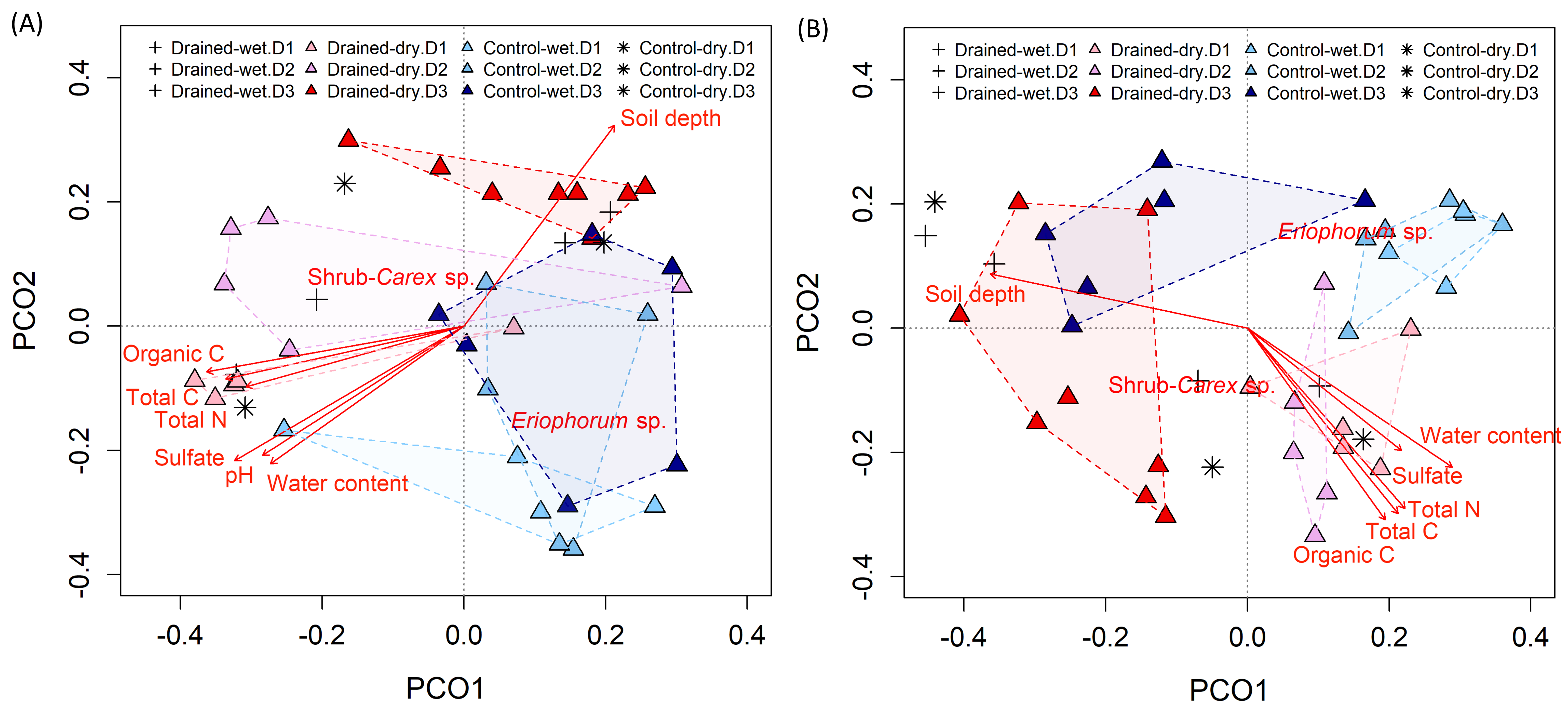


**Supplementary Figure 3. Principal coordinate analysis (PCoA) plots of (A) fungal and (B) non-fungal micro-eukaryotic communities fitted by vectors of soil physicochemical variables.** Only significant vectors and factors (goodness of fit, *R^2^* > 0.3 and *P* < 0.01) are displayed based on envfit analysis results.
